# Mortality in human sepsis is associated with downregulation of Toll-like receptor 2 and CD14 expression on blood monocytes

**DOI:** 10.1186/1746-1596-4-12

**Published:** 2009-04-16

**Authors:** Bernhard Schaaf, Karen Luitjens, Torsten Goldmann, Tobias van Bremen, Friedhelm Sayk, Christoph Dodt, Klaus Dalhoff, Daniel Droemann

**Affiliations:** 1 Medical Clinic Nord, Clinic Dortmund, 44145 Dortmund, Germany; 2 Medical Clinic III, University of Schleswig-Holstein, Campus Lübeck, 23538 Lübeck, Germany; 3 Clinical and Experimental Pathology, Research Center Borstel, 23845 Borstel, Germany; 4 Medical Clinic I, University of Schleswig-Holstein, Campus Lübeck, 23538 Lübeck, Germany; 5 Präklinik, Medical Clinic München-Bogenhausen, 81925 München, Germany

## Abstract

Pattern recognition receptors are a key component of the first line host defense against infection, recognizing specific microbial products. We hypothesize that monocyte hyporesponsiveness in human sepsis is associated with a downregulation of the pattern recognition receptors Toll-like receptor (TLR)-2 and TLR4.

Protein expression of CD14, TLR2 and TLR4 on blood monocytes was examined using flow cytometry from 29 patients with sepsis and 14 healthy controls. In addition LPS stimulated TNF-α and IL-10 production was studied in a 24 hour whole blood assay.

We found an increased expression of CD14, TLR2 and TLR4 in patients with sepsis compared to controls (p < 0.01). In patients with sepsis, death was associated with significant lower CD14 and TLR2 expression at admission (CD14: 25.7 +- 19.1 vs 39.1 +- 17.3 mean fluorescence intensity [MFI], p = 0.02; TLR2: 21.8 +- 9.4 vs. 30.9 +- 9.6, p = 0.01). At 72 hours the TLR2 expression on monocytes was associated with the IL-10 inducibility after LPS stimulation (r = 0.52, p = 0.02) and the CD14 expression with the IL-6, IL-10 and TNF inducibility.

We conclude that septic patients are characterized by an increased expression of CD14, TLR2 and TLR4 on monocytes compared to controls. Death is associated with downregulation of TLR2 and CD14 expression on monocytes correlating with reduced cytokine inducibility. We suggest that CD14 and TLR2 are a key factor in monocyte hyporesponsibility during severe sepsis.

## Background

Severe sepsis is the cause of 9% to 22% intensive care unit admissions and is associated with a mortality rate up to 50% [1]. Bacterial antigens trigger the initial cytokine response to infection, which is necessary for the clearance of invading pathogens, but overwhelming activation of immune cells, with excessive production of pro-inflammatory cytokines such as tumor necrosis factor (TNF)-α and interleukin (IL)-6, is thought to be responsible for the clinical manifestation of septic shock [2,3].

Recognition of pathogen associated molecular pattern (PAMP) by the innate immune system is mediated by pattern recognition receptors (PRR) on leucocytes and epithelial cells.

TLR4, together with CD14 and the MD2 adapter molecule, serves as the main receptor for components from gram negative bacteria such as lipopolysaccharide (LPS) [4], whereas TLR2 is crucial to the propagation of the inflammatory response to components mainly from gram-positive organisms, yeast and mycobacteria including lipoteichonic acid (LTA) and lipoarabinomannan [5-7]. In addition, TLR2 is activated by bacterial peptidoglycan, bacterial lipoproteins and lipopeptides, cell wall structures expressed on virtually all clinically relevant gram positive and gram negative bacterial pathogens [8]. TLR2 might also be involved in LPS induced cell activation since anti-TLR2 antibody partially inhibits IL-12 production of human dendritic cells [9]. TLR activation causes nulear factor kB translocation and mitogen-activated protein kinase phosphorylation, resulting in an enhanced production of inflammatory cytokines such as TNF, IL-1 and IL-6 [4]. In murine macrophages TLR4 expression correlates with the inducibility of the proinflammatory response to LPS [10], whereas stimulation with TLR2 agonist has been associated with the rapid release of IL-10 [11].

The central role of TLR2 and TLR4 in microbial responses suggests that they may be implicated in the pathophysiology and the outcome of human sepsis [12]. Beside initial systemic release of proinflammatory cytokines, prolonged cellular hyporesponsiveness to bacterial components with reduced cytokine response is thought to be a key factor in sepsis with limitation of subsequent ability to mount an appropriate inflammatory defense to secondary infections. TLR regulation might be implicated in hyporesponsiveness, since prolonged LPS-stimulation of human macrophages caused downregulation of TLR2 [13]. In addition tolerance in vitro to bacterial lipoprotein, a compound of gram-positive and gram-negative bacteria, is associated with reduced TLR2 expression [14].

In contrast to data describing TLR downregulation after LPS *in vitro*, murine sepsis is associated with an increased TLR4 protein expression in tissue [15]. In human sepsis increased protein expression and mRNA of TLR2 and TLR4 on blood neutrophils and monocytes are found compared to healthy individuals [16,17]. Thus, experimental models in animals and septic human patients display significantly upregulated TLR expression. But, in contrast to the resting state and nonseptic situation peripheral blood monocytes from septic patients secrete reduced quantities of proinflammatory cytokines regardless of their up regulated TLR-expression [18-21], possibly due to intracellulary inhibitory processes [22].

In addition to the questionable functional activity of TLR receptors during sepsis, expression of TLR during human sepsis was not associated with clinical outcome.

We hypothesized that TLR and CD14 expression is increased in sepsis, but inadequately less increased in severe sepsis compared to nonsevere sepsis, possibly associated to monocyte hyporesponsiveness.

We therefore compared the TLR2, TLR 4 and CD14 expression on blood monocytes of patients with sepsis and healthy controls. In patients with sepsis the TLR expression on monocytes was measured sequentially (admission, day 1, day 3, day 7) and correlated with sepsis severity and mortality. To evaluate the functional relevance of the receptor expression, we correlated the TLR and CD14 expression with the cytokine release after stimulation with LPS in a whole blood assay.

## Methods

### Sepsis patients

Twentynine patients with sepsis (defined according to 23) were investigated in a prospective manner. Patients below 18 years or with defined immunodeficiencies (hematologic or solid neoplasia, glucocorticoid or cytotoxic therapy, HIV infection or immunoglobulin deficiency) were excluded from the study.

The source of sepsis was the lung (n = 14), urinary tract (n = 8), meningitis (n = 2), gastrointestinal (n = 3), endocarditis (n = 1) and skin (n = 1). 28 von 29 (96,55%) patients had a predisposing chronic disease (pulmonary disease, cardiovascular disease, neurologic disease, renal insufficiency, diabetes mellitus).

### Healthy controls

14 unrelated healthy persons, all of white origin without signs of inflammatory disease served as a control group. The study was approved by the institutional ethics committee. Written informed consent was obtained from patients or their relatives and healthy volunteers.

### Study protocoll

Venous blood samples were obtained once in healthy controls and at admission (t0), 24 hours (t1), 72 hours (t2) and 7 days (t3).

### Sepsis severity

The disease severity was definded by the acute physiology score (APS), the APACHE II Score (including APS) and clinical/laboratory parameters. Septic shock was defined as sepsis associated with sepsis induced systolic blood pressure of < 90 mmHg for at least 30 min in the absence of other causes of shock, and at least 4 h of inotropic support after adequate fluid replacement were required [23].

#### PBMC purification and flow cytometry

30 ml of blood was obtained by venepuncture and collected into sterile heparinized tubes, PBMC were isolated by Bicoll/Ficoll density gradient centrifugation. PBMCs were cultured in 24-well tissue plates (Biochrome, Berlin, Germany) using endotoxin-free RPMI 1640 medium (Biowhittaker, Belgium) supplemented with 2 mM L-glutamine (Gibco, Eggenstein, Germany) at a density of 0.5 × 10^6 ^cells/ml at 37°C in a 5% CO_2 _humidified atmosphere for a period of 3 h. The expression of TLR2, TLR4 and CD14 on monocytes was determined using a fluorescence activated cell sorter (FACS Calibur, Becton Dickinson, Heidelberg, Germany). Data acquisition and analysis were performed with CellQuest software (Becton Dickinson, Heidelberg, Germany). Each measurement contained ≥ 10,000 cells in the monocyte population determined by characteristic forward/orthogonal light scattering in a density plot. Antibodies against the following epitopes were used. PE-labeled: TLR2, TLR4, CD14 isotype controls (eBioscience, San Diego, USA). PBMC (1 × 10^6^) were incubated on 4°C with 5 μl of anti-TLR2, -TLR4 PE monoclonal antibody respectively or isotype control. The expression of surface markers was calculated as mean fluorescence intensity (MFI) since no bimodal distribution was found.

#### Whole blood stimulation and cytokine assays

Whole blood stimulation assay was done as described previously [24]. In brief, 2,5 mL of heparinized blood was diluted 1:10 with RPMI 1640 (Biochrome, Berlin, Germany) supplemented with Pen/Strep 1% (Gibco, Germany) and immediately stimulated with 1 μg/mL LPS from Escherichia coli serotype 026:B6 (Sigma, St. Louis, USA) or with 100 μg/ml LTA from Stapylococcus aureus (Sigma L2515, St. Louis, USA) Samples were incubated in PPN tubes at 37°C with 5% CO_2_. Each experiment also included controls without LPS or LTA. Cell-free supernatants were removed after 24 h and stored at -80°C until assayed. Measurement of supernatant IL-6, TNF and IL-10 levels was performed using commercially available enzyme-linked immunosorbent assay kits, according to manufacturer's instructions (Biosource, Solingen, Germany).

### Statistics

Nonparametric statistics were used throughout the study. Data are given as mean ± SD. The Wilcoxon signed rank test was used for comparison of paired samples, for comparisons of independent samples the Mann-Whitney-U-test was used. Correlations were made with the Spearman's rank correlation. Calculations were carried out with Statistica for Windows, version 5, 1997. A p value of < 0.05 was considered significant.

## Results

### Patients

Demographic data of the study population are shown in table 1.

**Table 1:** Demographic and clinical data of 29 patients with sepsis.

age (Mean ± std.dev.) in years	68,55 ± 11,8
Male	14 (0,48)
APS	21,55 ± 7,50
APACHE II	30,86 ± 9,72
28 day mortality	8 (27,6%)
sepsis severity	
Sepsis	5 (17,2%)
severe Sepsis	5 (17,2%)
sept. Schock	12 (41,4%)
MODS	7 (24,1%)
septic complications	
acute renal failure	12 (41,4%)
DIC	7 (24,1%)
respiratory failure	19 (65,5%)

### TLR expression of sepsis patients compared to healthy controls

**Figure 1:**
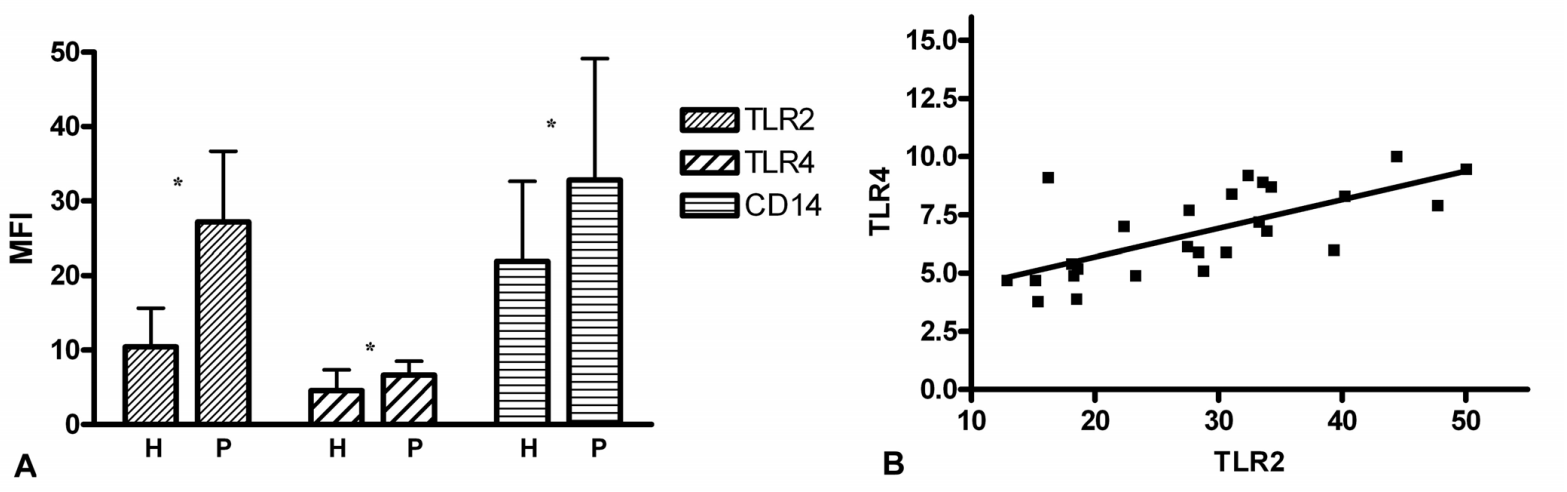
**A: Flow cytometry expression of TLR2, TLR4 and CD14 on monocytes**. MFI ± SD is shown from healthy volunteers (H) and patients with sepsis (P). * = p < 0.01 vs. controls. MFI = mean fluorescence intensity. **B**: Correlation between TLR2 and TLR4 in patients with sepsis (r = 0.69, p < 0.001).

The monocyte expression of TLR2, TLR4 and CD14 was at all timepoints [admission (t0), day 1 (t1) day 3 (t2) and day 7 (t3)] significantly higher in patients with sepsis compared to the monocyte expression in healthy controls (figure 1a). In healthy controls and in patients (at all time points) the TLR2, TLR4 and CD14 expression correlated significantly (figure 1b, p < 0.001 for all timepoints).

### TLR expression during the course of sepsis

**Figure 2:**
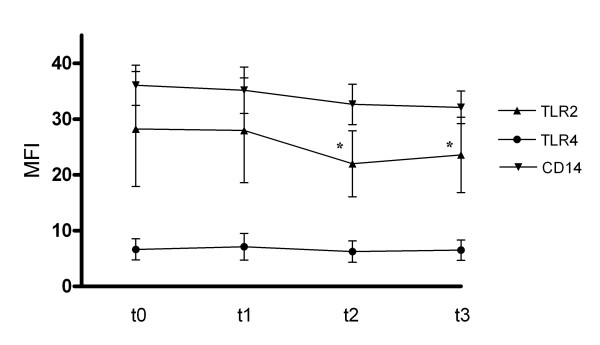
**Flow cytometry expression of TLR2, TLR4 and CD14 on monocytes during course of sepsis**. MFI ± SD is shown. * = p < 0.01 vs. t0. MFI = mean fluorescence intensity.

TLR-2 expression was downregulated between t0 and t3 (28.2 +- 10.3 vs. 23.8 +- 6.7 MFI; p = 0.01). CD14 was downregulated between t0 and t3 without reaching significance (35.1 +- 18.6 vs 31.6 +- 13.2; p = 0.07). TLR-4 was not regulated between t0 and t3 (6.6 +- 1.9 vs 6.5 +- 1.8; p = 0.35; figure 2).

### TLR expression according to sepsis severity

Death was associated with lower TLR-2, TLR-4 and CD-14 expression on blood monocytes, reaching significant values for TLR2 (at t0, t1 and t2) and CD-14 (at t0, table 2).

**Table 2:** TLR and CD14 protein expression on monocytes of patients with sepsis according to survival.

	Alive N = 21	Death N = 8	p
TLR 2			
T0	30.9 (+- 9.6)	21.8 (+- 9.4)	0.01
T1	30.5 (+- 7.6)	21.8 (+- 10.6)	0.02
T2	23.1 (+- 6.0)	18.7 (+- 4.5)*	0.04
T3	24.7 (+- 6.1)	20.4 (+- 8.6)**	0.3
TLR4			
T0	7.0 (+- 1.8)	5.7 (+- 2.0)	0.07
T1	7.4 (+- 1.8)	6.5 (+- 3.3)	0.08
T2	6.1 (+- 2.1)	6.5 (+- 1.3)*	0.9
T3	6.6 (+- 1.9)	6.1 (+- 1.8)**	0.6
CD14			
T0	39.1 (+- 17.3)	25.7 (+- 19.1)	0.02
T1	40.7 (+- 19.1)	24.7 (+- 18.0)	0.06
T2	34.2 (+- 20.2)	26.5 (+- 10.1)*	0.2
T3	31.3 (+- 12.5)	32.7 (+- 16.9)**	0.9

T0 = admission, T1 = 24 hours, T2 = 72 hours, T3 = 7 days. (Values as MFI), * n = 6, ** n = 5, less patients due to death

Patients with septic shock had a lower TLR-2 and CD-14 expression than patients without shock at t0, t1 and t2 without reaching significance (data not shown)

No correlation was found between TLR-2, TLR-4 or CD-14 expression and APS, APACHE II or Serum-CRP values (data not shown).

### Cytokine inducibility in whole blood stimulation assay

To test the cytokine inducibility and the hyporesponsiveness during sepsis, a whole blood assay with LPS *in vitro *stimulation was used.

Hyporesponsiveness was seen with lower IL-6, TNF and IL-10 inducibility at t0 compared with t3 without reaching significance (IL-6 t0: 3226 +- 4584 vs t3: 6563 +- 4923 pg/ml; p = 0.08, TNF t0: 664 +- 876 vs t3: 1278 +- 1234 pg/ml; p = 0.1, IL-10 t0: 20 +- 23 vs t3: 32 +- 22 pg/ml; p = 0.17).

**Figure 3:**
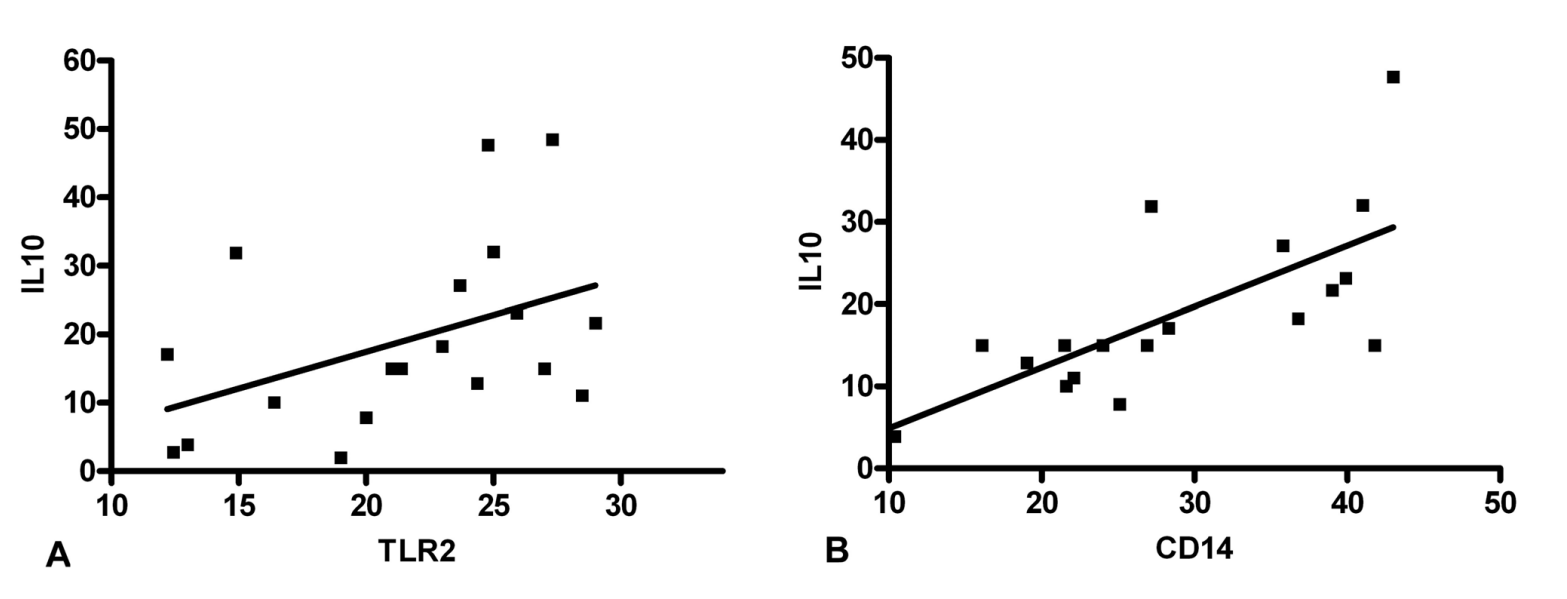
**Correlation between TLR2 (A, r = 0.52; p = 0.02, figure 3a), CD14 (B, r = 0.49; p = 0.04, figure 3b) and IL-10 secretion of whole blood in response to LPS-stimulation at t2 in patients with sepsis**.

To test the functional activity of TLR-2, TLR-4 and CD14, the cytokine inducibility after LPS stimulation was correlated with the receptor expression:

At t2, the TLR-2 expression was associated with the IL-10 inducibility (r = 0.52; p = 0.02, figure 3a) and the CD-14 expression was associated with the IL-6 (r = 0.49; p = 0.04), the IL-10 (r = 0.49; p = 0.04, figure 3b) and the TNF inducibility (r = 0.62; p = 0.03).

## Discussion

As shown by other investigators before, CD14, TLR2 and TLR4 protein expression on monocytes is elevated during sepsis. Interestingly we could show in our study a relatively lower receptor expression (CD14, TLR2 and as a trend in TLR4), in patients with sepsis that died, compared to patients that survived (Table 2). In addition we were able to demonstrate a correlation between receptor expression and cytokine production after LPS stimulation, leading to the assumption that the receptors on monocytes are functionally active during sepsis. These data underline the pathophysiologic role of TLR2 and CD14 for the activation of the immune system and the monocyte hyporesponsiveness in severe human sepsis.

As microorganisms trigger the release of cytokines via TLR's and CD14, these receptors are thought to play a central role in the pathophysiology of sepsis. The ability of the host to sense invasion of pathogenic organism and respond appropriately to control infection is critical to survival. Sepsis can induce monocyte hyporesponsiveness, limiting the antigen induced cytokine release causing immunosuppression. *In vitro *LPS stimulation is able to cause a downregulation of TLR2 and TLR4 gene expression and surface expression on mouse macrophages inducing monocyte hyporesponsiveness [25-27]. In contrast to downregulation of TLR gene expression after LPS exposure in cell culture, sepsis in mice [15] and humans sepsis causes an upregulation of TLR2, TLR4 and CD14 expression on blood monocytes (table 1). Similar data have been shown by other authors before [28,29]. Continuous microbial stimulation during bacterial sepsis with a number of different antigenic structures might result in cell activation inducing receptor upregulation [11]. Causative factors might be the release of cytokines during sepsis like IL-6, which has been shown to upregulate TLR4 on human monocytes [21]. In addition neutrophil products like elastase induces TLR4 expression on human monocytes [16].

In our study patients that died had lower cytokine inducibility at all timepoints and therefore more intensive hyporesponsiveness than patients that survived (n.s.). Correlating with this result, lower CD14 and TLR2 expression was found in patients that died (Table 2). In addition, at day 3 cytokine in vitro inducibility (24 hours whole blood LPS-stimulation) correlated with the TLR2 and CD14 protein expression on blood monocytes. Our data indicate that during the course of sepsis the receptors gain again functional activity, with higher cytokine release after in vitro antigen stimulation in cells with higher receptor expression. The data with lower receptor expression in patients that died during sepsis, confirm recent data, showing reduced gene expression for TLR receptors in monocytes of patients according to their state of sepsis: lowest TLR gene expression in septic shock, followed by severe sepsis and sepsis [30,31]. In addition to Salomao's gene results, we were able to associate TLR protein expression to sepsis severity. In conclusion, inadequate receptor activation might be deleterious for patients with sepsis.

A few years ago, TLR2 was thought to be a specific receptor for antigens from gram positive and the TLR2/CD14 complex was thought to be specific for gram negative antigenic structures. Several recent studies have shown that these receptors are not as specific as thought. Lipoteichonic acid (LTA) from *S. pneumonia *is able to activate TLR2 and CD14 [32]. In contrast LPS from gram negative bacteria is able to activate also CD14 and TLR2. The contribution of these receptors for host response during infection has been studied in animal knock out models. For example, TLR2 knock out causes reduced inflammatory response to pneumococcal LTA [32]. A strong pro-inflammatory reaction is necessary for local bacterial clearance. As shown in our study, an inadequate downregulation of TLR2 and CD14 (and consecutive reduced cytokine release) during sepsis might have a negative influence on outcome. In addition to reduced bacterial clearance, monocyte hyporesponsiveness has been associated with increased number of nosocomial infections in septic patients. Beside innate immune response, the TLR2 induced cytokine response might also be involved in the physiologic endocrine response to sepsis, since TLR2 deficient mice have an impaired cytokine mediated corticosteroid adrenal stress response [33]. Taken together, these data suggest that a reduced TLR2 and CD14 expression during sepsis might have a negative outcome in sepsis.

Therapies targeting TLR-pathways are currently being investigated for several diseases. Whether TLR blocking [34] or TLR agonists [35] are needed in sepsis is under discussion. Broad knowledge about the regulation of TLR and CD14 induced signalling pathways are needed to anticipate the clinical effect. Our data suggest that additional TLR blocking in patient with sepsis might be dangerous.

## Conclusion

In conclusion septic patients are characterized by an increased TLR2, TLR4 and CD14 expression. Death due to sepsis is associated with TLR2 and CD14 downregulation. The precise role of TLR and CD14 regulation in sepsis should be evaluated in further clinical and experimental studies, especially before TLR blocking agents are clinically investigated.

## Competing interests

The authors declare that they have no competing interests.

## Authors' contributions

KL and TB carried out the flow cytometry and were involved in the design of the study and drafting the manuscript. TG carried out cell culture experiments and was involved in drafting the manuscript. BS, FS, CD, KD and DD conducted the clinical part of the study and were involved in the design and coordination of the study and drafting the manuscript. All authors read and approved the final manuscript.
